# Gastric Perforation Secondary to Acute Gastric Dilatation Without an Eating Disorder: A Case Report

**DOI:** 10.7759/cureus.93513

**Published:** 2025-09-29

**Authors:** Abderrahman Atmani, Mokhtar Abdelmalek, Anass El Amrani, Enrico Volpin

**Affiliations:** 1 Surgical Oncology, Mohammed VI University Hospital Center/Mohammed First University, Oujda, MAR; 2 Surgery, Simone Veil Hospital, Eaubonne, FRA; 3 Surgical Oncology, Mohammed VI University Hospital Center, Regional Oncology Center, Oujda, MAR; 4 Gastrointestinal Surgery, Simone Veil Hospital, Eaubonne, FRA

**Keywords:** acute gastric dilatation, eating disorders, gastrectomy, gastric perforation, witzel feeding jejunostomy

## Abstract

Acute gastric dilatation (AGD) is a life-threatening condition that could progress to necrosis and perforation. Most cases are associated with mechanical obstruction or eating disorders; however, spontaneous perforation after a single large meal in patients without prior risk factors is exceptionally rare.

A 64-year-old woman with no history of eating disorders was admitted with peritonitis and septic shock, 24 hours after consuming a large meal. Computed tomography showed pneumoperitoneum, free intra-abdominal fluid and a gastric perforation. Emergent laparotomy revealed necrosis and a 6 cm perforation in the anterior gastric wall, requiring partial gastrectomy and feeding jejunostomy. Despite severe initial acidosis and acute kidney injury, the patient recovered fully after 10 days of intensive care. Histopathology confirmed ischemic necrosis without evidence of microorganisms or malignancy.

This case highlights that a gastric necrosis caused by AGD can occur even in the absence of classic risk factors. Venous ischemia from intraluminal pressure elevation - rather than arterial insufficiency - was the likely mechanism. Early recognition and surgical intervention are critical to survival.

## Introduction

Acute gastric dilatation (AGD) is a rare condition characterized by extreme expansion of the stomach. In most cases it’s due to mechanical obstruction, eating disorders, or non-mechanical causes such as acute pancreatitis, diabetic gastroparesis, electrolyte disturbances, and spinal pathology [1,2]. AGD could lead to gastric necrosis and perforation [1,3].

Gastric perforation secondary to AGD is in most cases attributed to factors such as anorexia nervosa, pyloric stenosis or postoperative complications [1,2,4-6]. Spontaneous necrosis following a single large meal in patients without prior eating disorders - as in our case - is rare. The pathophysiology involves transmural infarction caused by venous ischemia due to elevated intraluminal pressure [7,8].

Herein we report the case of a 64-year-old woman with no prior history of eating disorders who developed gastric necrosis, perforation, and septic shock after a probable binge eating episode, requiring emergent laparotomy and partial gastrectomy. This case report highlights the importance of early recognition of this entity, the role of computed tomography in diagnosis, and the prompt surgical management to reduce mortality.

## Case presentation

**Figure 1:**
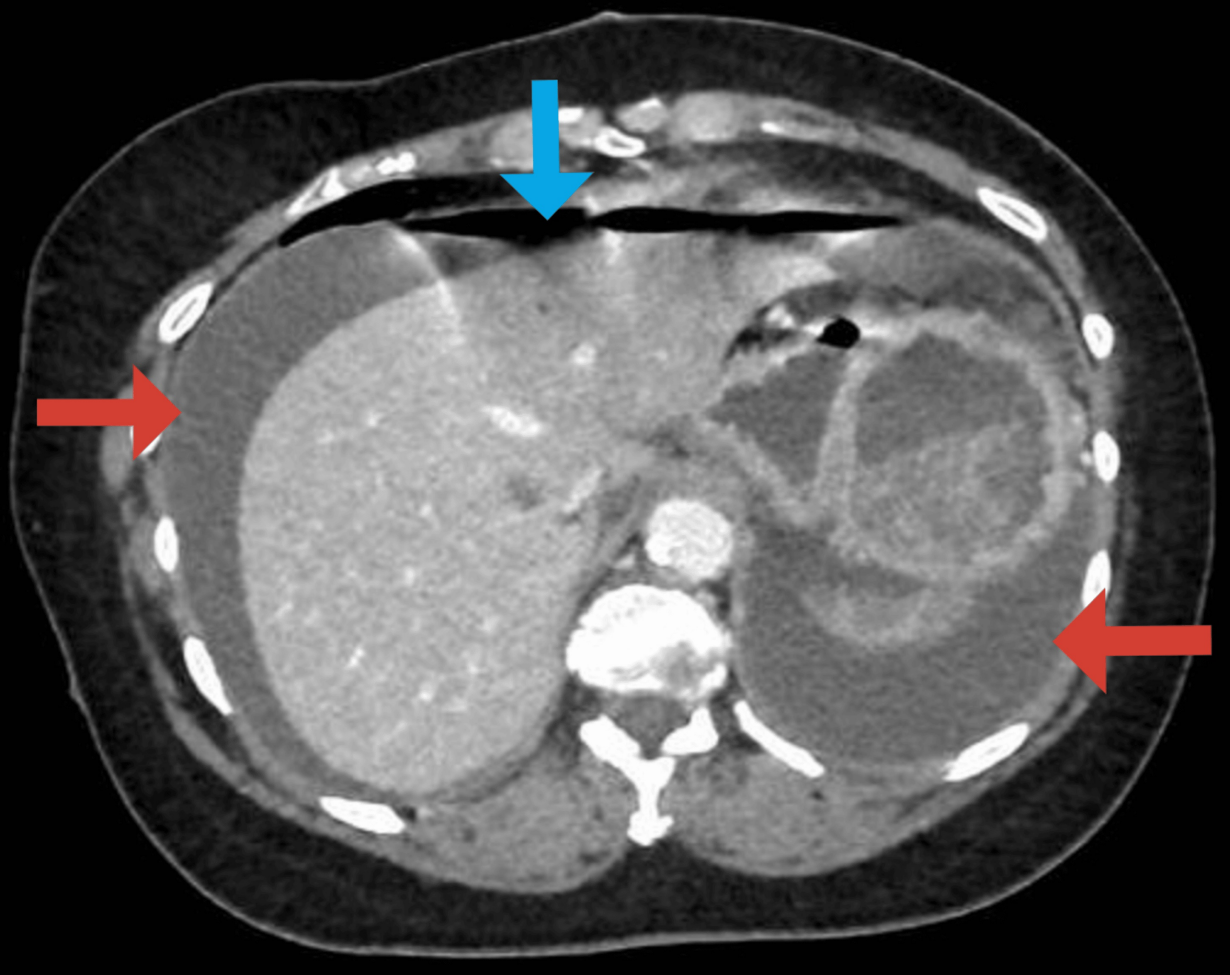
Contrast-enhanced abdominal computed tomography revealing pneumoperitoneum (bleu arrow) and significant peritoneal effusion (red arrows).

**Figure 2:**
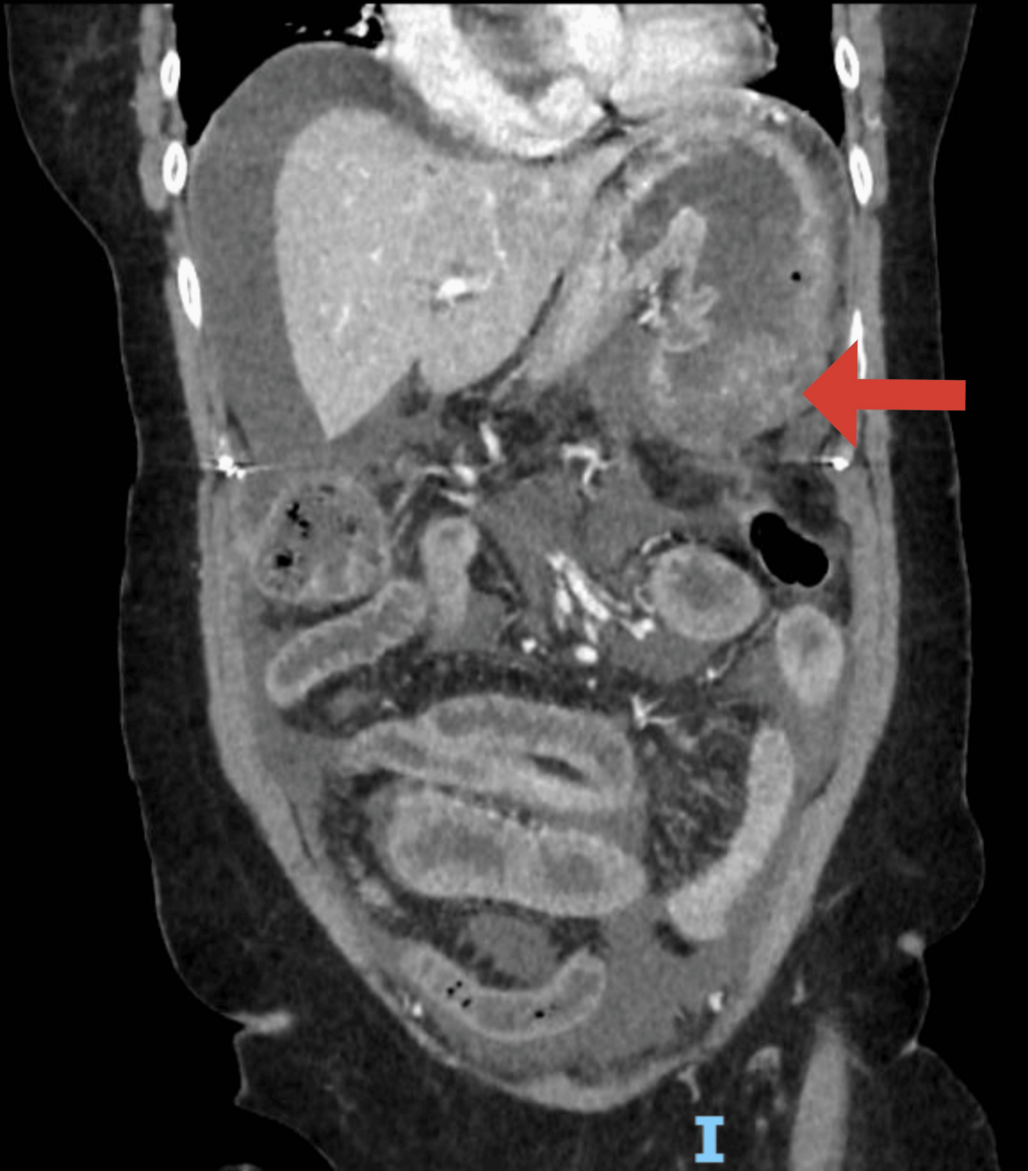
Contrast-enhanced abdominal computed tomography scan demonstrating the site of the gastric wall disruption (red arrow).

A 64-year-old woman with a history of ascending aortic aneurysm and no history of an eating disorder. She had no other significant medical history, including no history of eating disorders, other comorbidities, or regular medication use. She was admitted to the emergency department. She presented generalized abdominal pain, started 24 hours earlier, and alimentary vomiting after a large meal consisting of rice, vegetables, and 1 liter of sparkling water. She was asthenic, in severe pain, with oxygen saturation at 78%, tachycardia at 150 beats per minute, blood pressure of 110/75 mmHg, generalized mottling, and distended abdomen. On physical examination, she was afebrile, had tenderness and involuntary guarding in all abdominal quadrants. The patient was immediately resuscitated. Double-wide bore IV lines were maintained. IV fluids, antibiotics, and analgesics were administered. Lab tests revealed the following: total leukocyte count was 9,600/μL, hemoglobin level was 19 g/dL, and hematocrit was 57%. C-reactive protein was elevated at 53 mg/L (normal: <5 mg/L). Acute kidney injury was present, with blood urea at 12.9 mmol/L (normal: 2.9-8.2 mmol/L) and creatinine at 209 µmol/L (normal: 45-84 µmol/L). Glucose was 12.00 mmol/L (normal: 4.11-5.89 mmol/L). Sodium was 150 mmol/L (normal: 136-145 mmol/L). Potassium was 3.6 mmol/L (normal: 3.5-4.5 mmol/L). Chloride was 102 mmol/L (normal: 97-108 mmol/L). Bicarbonates were 20 mmol/L (normal: 22-29 mmol/L). Proteins were 99 mmol/L (normal: 64-83 mmol/L). Inonic gap was 32 mmol/L (normal: 14-18 mmol/L). Arterial blood gas analysis showed an elevated lactate at 4.5 mmol/L (normal: <2 mmol/L) with significant acidosis (pH = 7.03). A CT of the abdomen and pelvis was performed after stabilization of the patient, revealing a large volume pneumoperitoneum, significant peritoneal effusion, and a focal discontinuity in the anterior gastric wall, likely representing the site of perforation. No occlusion of the celiac-mesenteric arterial system was noted (Figures 1, 2).

**Figure 3:**
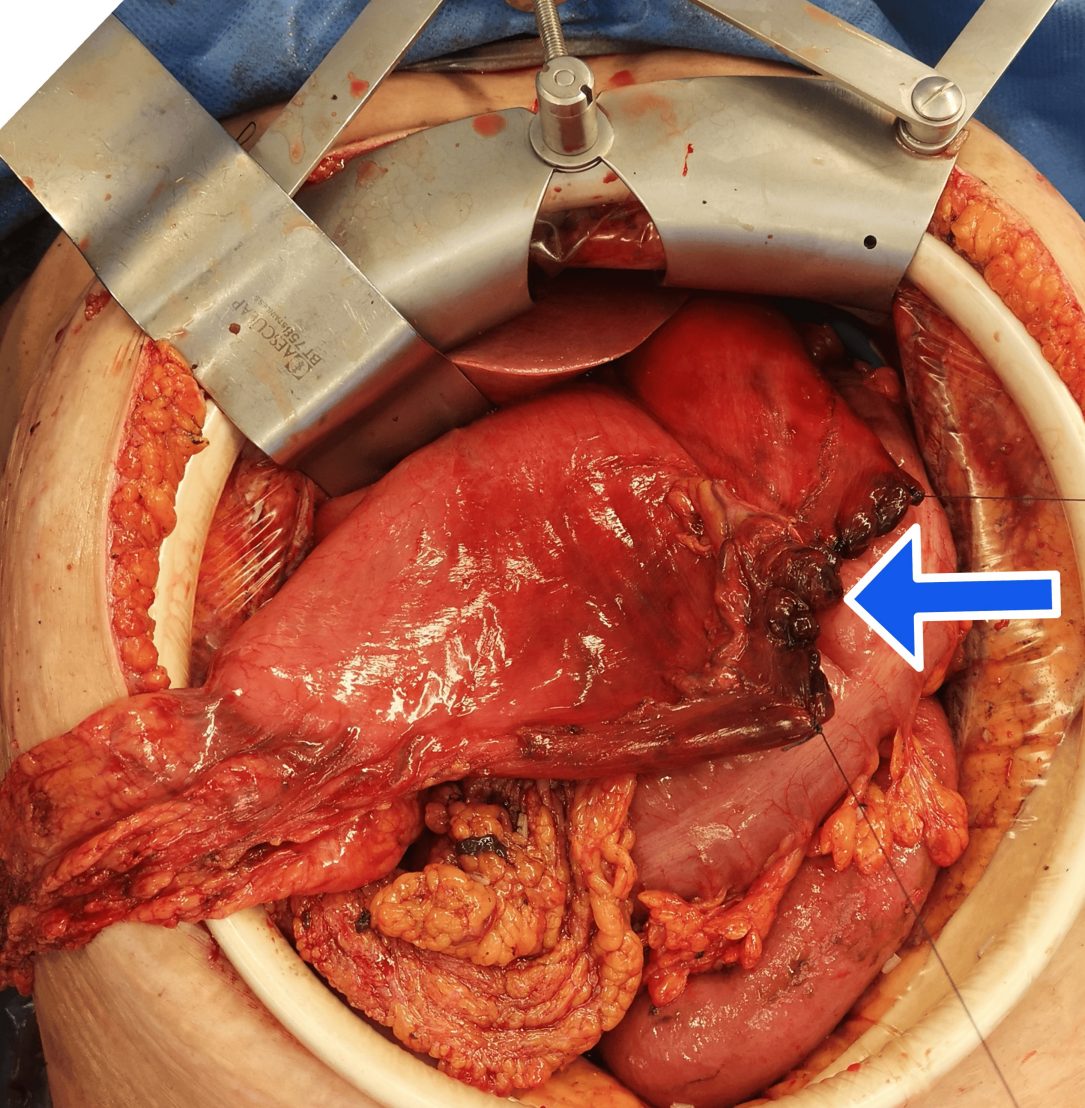
Intraoperative view showing the necrotic gastric perforation site with temporary closure (Blue arrow)

**Figure 4:**
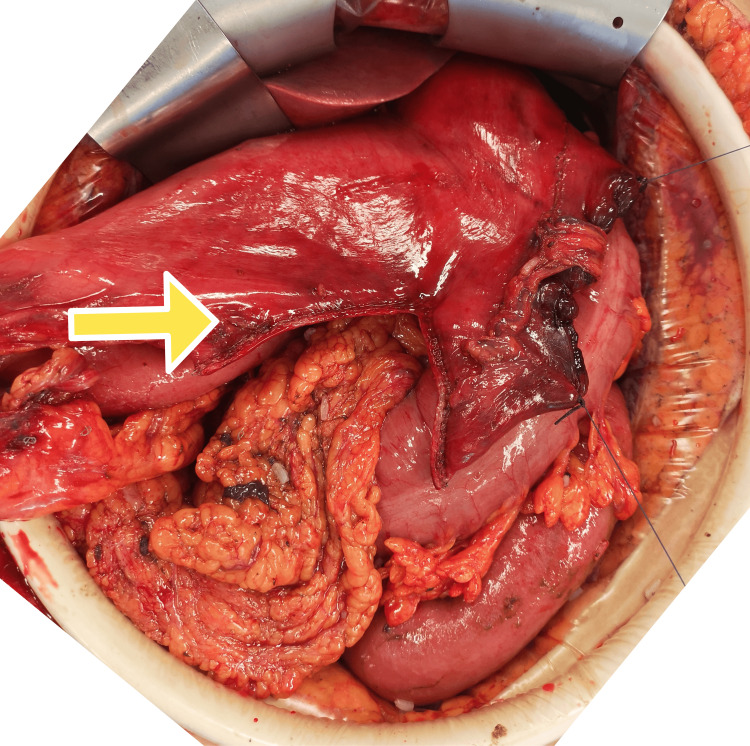
Intraoperative view of the initiation of the sleeve-like gastrectomy. The yellow arrow indicates the initial staple line of the resection.

Hence, an immediate xypho-supra-umbilical exploratory laparotomy was performed. A large perforation surrounded by necrotic changes was found on the greater curvature of the stomach, while the esophagus and duodenum did not show any abnormalities. A temporary closure of the perforation was performed (Figure 3), and 3 liters of digestive fluid with food debris were aspirated from peritoneal cavity. Given the extent of necrosis, a vertical partial gastrectomy was performed using a 65 mm linear stapler with a blue cartridge (Figure 4). The abdominal cavity was thoroughly irrigated to remove residual inflammatory fluid and prevent further contamination. A Witzel feeding jejunostomy was created on the first jejunal loop. Two drains on suction were placed: one in the pelvis and a second drain along the right parietocolic gutter for postoperative monitoring and drainage. The procedure was completed in two hours and 20 minutes, with an estimated blood loss of 35 mL.

**Figure 5:**
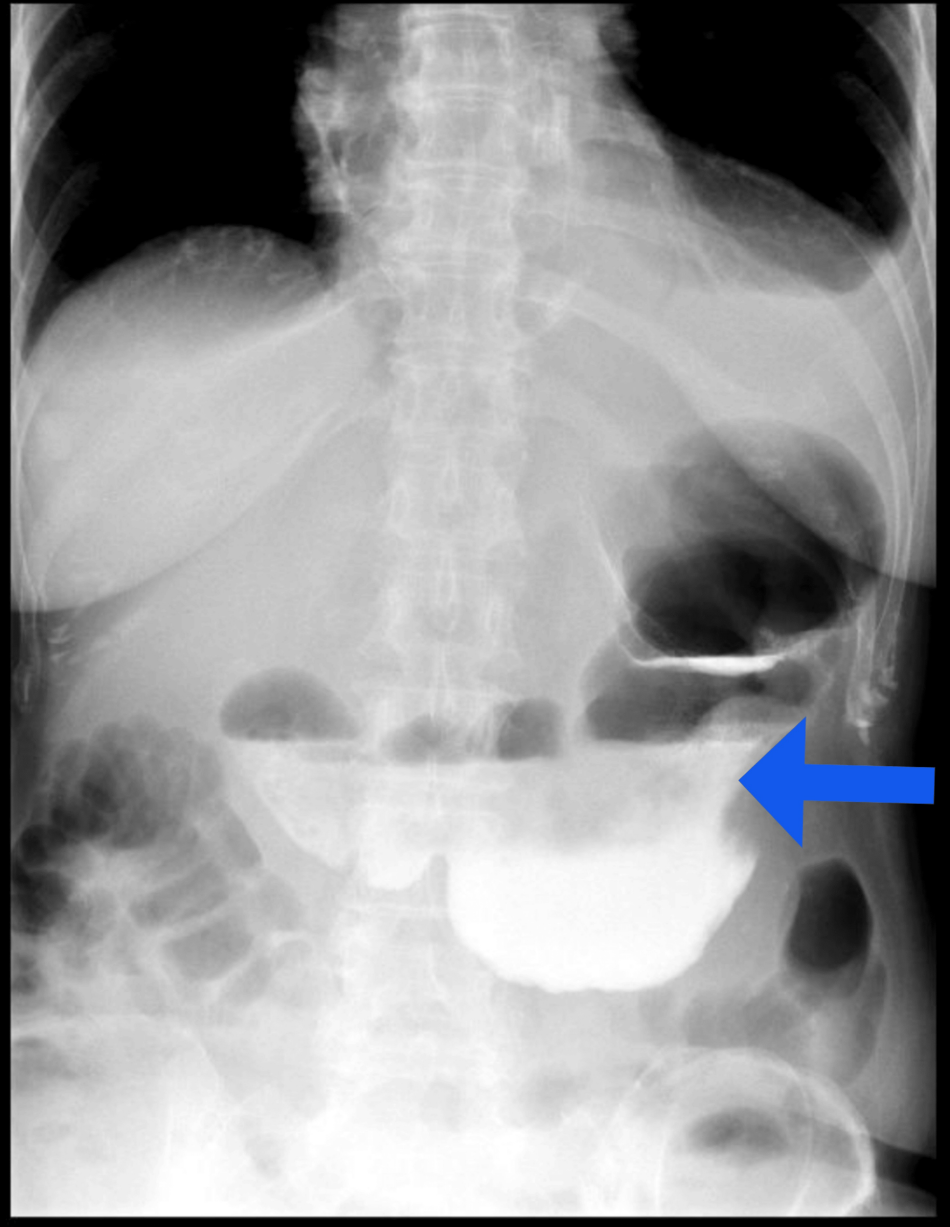
Normal opacified abdominal radiograph confirming adequate postoperative function (blue arrow).

A nasogastric tube was placed in low suction. The patient remained in the critical care setting for 10 days due to septic shock and acute pulmonary edema. Enteral nutrition via the jejunostomy was initiated on postoperative day two. After returning to the general surgery service, the patient was started on a soft diet, which was well tolerated, and she was allowed home on postoperative day 11. Prior to discharge, an opacified X-ray and CT scan demonstrated normal findings with adequate gastric emptying (Figure 5). No weight loss or nutritional deficiency was observed on the follow-up. Histopathological examination reported diffuse ischemic changes without evidence of microorganisms or malignancy.

## Discussion

AGD was reported for the first time by S.E. Duplay in 1833. It could be secondary to mechanical obstruction ranging from pyloric stenosis to gastric volvulus, or extrinsic compression such as superior mesenteric artery syndrome. Non-mechanical causes such as diabetes mellitus, eating disorders such as bulimia or anorexia nervosa, postoperative complications, electrolyte imbalances, or medication effects [4-7]. Gastric necrosis is rare due to the stomach’s rich blood supply [1,3,8,9].

Although rare, gastric ischemia secondary to AGD is a severe complication caused by increased intraluminal pressure, which is a critical factor for perforation [1]. In AGD, ischemia develops when intraluminal pressure surpasses venous pressure, leading to submucosal venous occlusion and mucosal hypoperfusion. Progression may cause venous thrombosis, infarction, and perforation [1,9-12]. We report a rare case of gastric necrosis, perforation, and shock resulting from AGD likely due to a binge-eating episode, despite no prior history of an eating disorder. In our case, the perforation was in the anterior gastric corpus. These ischemic lesions typically occur near anastomoses between the arterial arches from the lesser to greater curvature along the anterior or posterior walls [9]. On the other side, it is also thought that the stretching compliance of the lesser curve is countered by its anatomical fixation and a lesser number of mucosal folds [8,10,11].

Prompt diagnosis of AGD is critical, as timely intervention significantly improves survival outcomes [1]. In the absence of peritonitis, nasogastric decompression could lower intraluminal pressure and prevent necrosis [12]. In our case, perforation was present on admission with peritoneal signs and altered general status, making urgent surgical intervention obligatory. The surgical management involves resection of necrotic tissue, requiring partial or total gastrectomy. Concurrent feeding jejunostomy placement is advised given the anticipated prolonged nil per os status during recovery [1,4,9,12].

This case highlights the need for suspicion of gastric necrosis in patients with acute abdominal pain after large meals, even without a history of eating disorders.

## Conclusions

This case reinforces the necessity of including AGD-induced necrosis in the differential diagnosis for severe abdominal pain, even in the absence of traditional risk factors. It elucidates a venous ischemic mechanism and demonstrates that the condition can arise following a single binge eating episode. The critical determinants of survival are timely diagnosis, vigorous resuscitation, and urgent surgical intervention. Collecting further data on these non-classical presentations is crucial to refining diagnostic criteria and therapeutic strategies.
